# *Amomum tsao-ko* volatile oil alleviates renal fibrosis in 5/6 nephrectomy rats through TGF-*β*1/SMAD3 and PI3K/AKT signaling pathways

**DOI:** 10.3389/fphar.2026.1819932

**Published:** 2026-10-08

**Authors:** Huanyue Cui, Meiru Shi, Qing Xu, Shangwen Zhou, Xin Li, Hangyu Wang, Dahong Yao, Ke Zhang, Pinghua Sun, Jinhui Wang

**Affiliations:** 1 School of Pharmacy/Key Laboratory of Xinjiang Phytomedicine Resource and Utilization, Ministry of Education/Institute for Safflower Industry Research/School of Chemistry and Chemical Engineering, Shihezi University, Shihezi, China; 2 School of Pharmaceutical Sciences, Shenzhen Technology University, Shenzhen, China; 3 International Cooperative Laboratory of Traditional Chinese Medicine Modernization, Innovative Drug Development of Chinese Ministry of Education (MOE), College of Pharmacy, Jinan University, Guangzhou, China; 4 Department of Medicinal Chemistry and Natural Medicine Chemistry (State-Province Key Laboratories of Biomedicine-Pharmaceutics of China), Harbin Medical University, Harbin, China; 5 Xinjiang Academy of Forestry, Key Laboratory of Forest Resources and Utilization in Xinjiang of National Forestry and Grassland Administration, Urumqi, China; 6 Xishuangbanna Dai Medicine Research Institute Co., Ltd., Jinghong, China

**Keywords:** *Amomum tsao-ko* volatile oil, renal fibrosis, 5/6 nephrectomy, TGF-*β*1/SMAD3 signaling pathway, PI3K/AKT signaling pathway, transcriptomics

## Abstract

**Introduction:**

Renal fibrosis is the main cause of many kidney diseases and a common outcome of many chronic kidney diseases. The dried mature fruit of *Amomum tsao-ko* is widely used to treat various diseases, including those characterized by diuresis and detumescence. *Amomum tsao-ko* volatile oil (AO) may be the active ingredient responsible for its main pharmacological action, and its protective effect and mechanism in kidney injury models are unclear. Therefore, based on transcriptomics and animal experiments, this study discusses the mechanism of AO through TGF‑*β*1/SMAD3 and PI3K/AKT signaling pathways in renal fibrosis of 5/6 nephrectomy rats.

**Methods:**

Seventy-two healthy SD rats were randomly divided into 6 groups. The rat renal fibrosis model was established by 5/6 nephrectomy. Different doses of AO were given by gavage. After 28 days of continuous administration, the levels of creatinine and urea nitrogen in serum of rats were detected. The total RNA of rat kidney tissue was extracted for transcriptomics determination and analysis showed the expression of differential genes in each group. The core targets related to chronic kidney injury were docked with the main components of the AO. HE, PAS and Masson staining were used to observe the pathological morphology of renal tissue in rats. Western Blot was used to detect the expression of p-PI3K, PI3K, AKT, p-AKT, SMAD3, TGF-*β*1, MMP-9 and TIMP-1 in renal tissue.

**Results:**

Compared with the Sham group, the transcriptome results showed that there were 823 genes with significant differences in expression; the levels of SCr and BUN in the model group were significantly increased. HE, PAS and Masson found that 5/6 Nx could develop interstitial fibrosis. At the same time, a large number of renal tubular epithelial cells were atrophied, deformed and exfoliated, and a large number of inflammatory cells infiltrated in the interstitium. Western Blot results showed that the expression of PI3 K, AKT, TIMP-1, SMAD3, TGF-*β*1 and other proteins in the 5/6 Nx group increased significantly, and the expression of MMP-9 protein decreased significantly. Compared with the 5/6 Nx group, there were 1,009 genes with significant differences in expression after treatment with AO, and the levels of SCr and BUN decreased. The infiltration of inflammatory cells in renal tissue was improved. The expression of PI3K, AKT, TIMP-1, SMAD3 and TGF-*β*1 was significantly decreased, and the expression of MMP-9 was significantly increased.

**Conclusion:**

AO can further inhibit the phosphorylation level by inhibiting the TGF- *β*1/SMAD3 and PI3K/AKT signaling pathways in 5/6 Nx rats, thus effectively improving the pathological damage of renal tissue.

## 1 Introduction

Renal fibrosis is a common pathological process in the development of chronic kidney disease (CKD) to end-stage renal disease (Gan et al., 2025). It is characterized by excessive deposition of extracellular matrix (ECM) and destruction of renal tissue structure, leading to the gradual loss of renal function (Sharma et al., 2025). Progressive renal fibrosis is the central pathological feature of chronic kidney disease (CKD), which imposes an enormous worldwide disease burden. The GBD 2023 systematic analysis published in The Lancet GBD (2023) reported 788 million adult CKD cases globally in 2023, with an age-standardized prevalence up to 14.2%. CKD-related deaths have reached 1.48 million per year, largely attributable to unimpeded glomerular and tubular fibrosis advancing to ESKD. Angiotensin-converting enzyme inhibitors (ACEI) and angiotensin II receptor antagonists (ARB) are the most widely used core anti-fibrosis drugs with the most sufficient clinical evidence (Huang et al., 2025; Kim et al., 2025). However, they cannot directly block myofibroblast activation and ECM deposition, which can only delay disease progression, and are expensive and bring heavy medical burden to patients (Shi et al., 2026). In the early stage of renal fibrosis, inflammatory cells such as lymphocytes and monocytes are infiltrated and damaged, producing pro-fibrotic factors such as TGF-*β*1, which activate fibroblasts and induce their proliferation and differentiation into myofibroblasts (Lee et al., 2024; Bai et al., 2019). Renal fibrosis is mainly driven by α-smooth muscle actin (α-SMA)-positive myofibroblasts. Myofibroblasts are the main source of ECM (Bouguéon et al., 2024), and their abnormal activation plays an important role in renal fibrosis. Therefore, most anti-fibrosis drug mechanisms are blocking the activation of myofibroblasts.

AO was extracted by steam distillation. The main components of the AO were analyzed by GC-MS technology. A total of 56 components were identified, mainly including eucalyptol (46.42%), (E)-citric acid (5.14%), geraniol and so on (Xu et al., 2022). *Amomum tsao-ko* Crevost et Lemarie is a dry and mature fruit of *Amomumtsao-ko*, which is widely distributed in southwest China and other places (Guo et al., 2025). It was first recorded in Compendium of Materia Medica “ as ” treating edema and diarrhea, collapsing in the middle and bringing down (Jia et al., 2020). It has the effects of warming stomach and relieving pain, drying dampness and invigorating spleen, diuresis and detumescence (Wang et al., 2025). Its special aroma from AO has been extensively studied by domestic experts and scholars in recent years. Through extraction and separation, structural identification and activity research, it has been found that the AO has antioxidant, antibacterial, anti-inflammatory, anti-tumor and other pharmacological effects (Xu et al., 2022). Natural products have become an important alternative source for the treatment of kidney diseases. As a homologous species of medicinal food, *Amomum tsao-ko* has mild medicinal properties, high safety and long-term use. Based on traditional experience and multi-target characteristics, *Amomum tsao-ko* has great research prospects in the prevention and treatment of kidney diseases.

Based on this, the purpose of this study is to use modern pharmacological methods to scientifically verify the protective effect of AO, a key component of traditional drugs, on renal function injury, and to explore its potential mechanism of action. This not only helps to clarify the scientific connotation of traditional experience but also provides an experimental basis for the development of new strategies for kidney protection based on ethnic medicinal plants.

## 2 Materials and methods

### 2.1 Experimental animal

Estrogen in female rat can relieve renal fibrotic injury and cause unstable pathological phenotypes, which may increase experimental variation. To eliminate hormonal interference and ensure uniform lesion severity among groups, male rats were selected, consistent with most related CKD studies. Seventy-two clean-grade male SD rats, weighing 200–220 g, aged 7–8 weeks, were purchased from the Animal Experimental Center of Xinjiang Medical University, License No. SCXK 2018-0003. They were housed at a feeding temperature of 23–26 °C and humidity of 40%–70% and exposed to a 12:12-h light-dark cycle, with free access to food and water. All of the experimental procedures were performed in accordance with the Animal Management Guidelines for the Experimental Animal Center of the School of Pharmacy, Shihezi University, and the experimental protocols were previously approved by the Ethics Committee of Shihezi University Affiliated Hospital.

### 2.2 Medicine and reagent

In this study, *Amomum tsao-ko* Crevost et Lemaire was purchased from Anguo Shengshan Pharmaceutical Co., Ltd., and identified by Professor Zhu Yun of Shihezi University as the dried fruit of *Amomum tsao-ko*.

Extraction and solution preparation of volatile oil from *Amomum tsao-ko*. The dried fruits of *Amomum tsao-ko* were crushed into coarse powder. The volatile oil was extracted by steam distillation according to the method described in the 2020 edition of the Chinese Pharmacopoeia (General Chapter 2204, Method A). The coarse powder was placed in a 1,000 mL round-bottom flask, and distilled water was added from the upper end of the condenser until the graduated portion of the volatile oil extractor was completely filled. After soaking for a specified period, the mixture was heated using an electric heating mantle and distilled for a defined duration. The resulting yellow oily substance was collected as the volatile oil of *Amomum tsao-ko*. The oil was stored in a brown glass bottle at 4 °C in the dark. The extraction yield was calculated as follows: Yield (%) = [Volume of volatile oil (mL)/Weight of dried material (g)] × 100%.

After the volatile oil of *Amomum tsao-ko* was extracted, it was mixed according to the volatile oil: Tween-80 = 1:1 (volume ratio) before use, gently ground evenly, and then diluted with 0.1% Tween-80 aqueous solution to the required concentration, and ultrasonically treated for 10 min to form a uniform suspension.

Urea nitrogen, urine protein, creatinine and other kits were purchased from Nanjing Jiancheng Bioengineering Institute (Nanjing, China). PI3K, P-PI3K, AKT, p-AKT and other primary antibodies were purchased from Boster Biotechnology Co., Ltd., and *β*-actin was purchased from Beijing Zhongshan Jinqiao Biotechnology Co., Ltd.

### 2.3 Model establishment and experimental design

After 1 week of acclimation, the animals were randomly assigned to either Sham group or 5/6 Nx group. To establish a rat model of CKD, the 5/6 Nx group underwent in two stages. The sham control group underwent a two-stage open surgery without nephrectomy.

The surgical process was as follows: in the first stage, fasted and water deprived for 8 h before surgery, anesthetized rats with pentobarbital sodium, and fixed the rats in a lateral position on the surgical plate. The left dorsal hair was removed, the skin was prepared, and routine disinfection was performed. A longitudinal skin incision was made at the left rib ridge angle of 1.0 cm, and the skin layers were separated layer by layer into the posterior abdominal cavity. After peeling off the renal capsule and adrenal gland, clamp the renal pedicle with hemostatic forceps and quickly remove the upper and lower poles of the left kidney, with a total removal amount of about 2/3 of the left kidney. Gelatin sponge pad was used to compress and stop bleeding. After observation of no active bleeding, the left kidney was reset, and the incision was sutured layer by layer and bandaged and disinfected. Postoperative intramuscular injection of penicillin 720,000 U/kg.

One week after the first surgery, a second total nephrectomy is performed: anesthesia, fixation, skin incision, and separation of the right kidney are performed in the same manner as the previous first surgery. After peeling off the renal capsule and adrenal gland, clamp the renal pedicle with hemostatic forceps, ligate the renal pedicle with sutures, and then remove the right kidney. Suture the incision and feed separately. On the day after surgery, inject 720,000 U/kg penicillin intramuscularly once a day for three consecutive days.

After the 5/6 nephrectomy, rats in the 5/6 Nx group were randomly assigned to five groups: 5/6 Nx group, 5/6Nx + AO 40 mg/kg, 5/6Nx + AO 80 mg/kg, 5/6Nx + AO 160 mg/kg and 5/6Nx + LOS 10 mg/kg (Losartan was used as a positive control drug, and its dose (10 mg/kg/d) was based on the effective dose of renal fibrosis model reported in previous literature. This dose has been shown to significantly reduce blood pressure and improve renal function indicators.) (Zou et al., 2022; Zhang et al., 2026) by oral gavage once per day for 28 days. The sham and 5/6Nx groups received the same amount of 0.1% tween-80.

In this study, 72 SD rats were initially enrolled, including 12 in the sham operation group, and the remaining 60 underwent 5/6 nephrectomy. Within 3 days after operation, there was no death in the sham operation group, and 8 died of infection in the operation group. From the fourth day, the surviving rats were randomly divided into 5 groups for drug treatment. By the end of the experiment on the 28th day, 11 rats died in succession, 12 rats survived in the sham operation group, and at least 8 rats survived in each group of the remaining groups.

### 2.4 Detection of serum biochemical indexes and quantitative detection of urine protein

The levels of BUN and SCr in serum of rats were detected, and the level of 24 h UTP was detected by urine analyzer. according to the manufacturer’s instructions.

### 2.5 Renal histopathological examination

The renal tissue was fixed with 4% paraformaldehyde, dehydrated by ethanol, embedded in paraffin and sectioned. After dewaxing and dehydration, the sections were stained with hematoxylin and eosin (H&E), periodic acid-Schiff (PAS) and Masson, and subsequently observed under light microscope for renal histopathological changes. The semi quantitative scoring of glomerulosclerosis in PAS stained glass slides and interstitial collagen deposition in Masson stained glass slides was calculated using the method described in the literature: 10 views were randomly selected under light microscopy, and the lesion area was scored according to the following criteria (0, <10%; 1, 10%–25%; 2, 25%–50%; 3, 50%–75%; 4, 75%–100%).

### 2.6 RNA-seq analysis

The renal tissues of rats in the sham operation group, the model group and the high-dose *Amomum tsao-ko* group were taken 50 mg each, and grinded and broken after quick freezing in liquid nitrogen. The total RNA of each group was extracted and the quality was detected by Agilent 2,100 Bioanalyzer. Appropriate amount of total RNA was taken, and mRNA was fragmented after DNase I treatment. The fragmented mRNA was used as a template to synthesize the first-strand cDNA, and then the double-strand cDNA was synthesized. The double-stranded cDNA was subjected to terminal repair, ligation of double exponential adaptor, chain selection and 15 cycles of PCR amplification to complete the construction of RNA library. The library concentration and fragment size were detected by Agilent 2,100 Bioanalyzer, and the library was sequenced on the MiniSeq platform after equal molar collection. After sequencing, the library was recombined and purified according to the index balance.

### 2.7 Real-time quantitative PCR analysis (RT-qPCR)

Total RNA was extracted from kidney tissue using Trizol reagent (ET101, TransGen Biotech Co., Ltd., Beijing, China), and RNA concentration was measured with a Q5000 (Quawell Technology, Inc., United States). cDNA was synthesized using a reverse transcription kit (AT311, TransGen Biotech Co., Ltd., Beijing, China) according to the manufacturer’s instructions. RT-qPCR was performed using an RT-qPCR kit (AQ601, TransGen Biotech Co., Ltd., Beijing, China) and relative expression was calculated using the 2–ΔΔCT method. Primer sequences are listed in Table 1.

**TABLE 1:** qPCR primer sequences.

Primer	Sequence ((5′to 3′)
GAPDH (rat) -F	ACG​GCA​AGT​TCA​ACG​GCA​CAG
GAPDH (rat) -R	CGA​CAT​ACT​CAG​CAC​CAG​CAT​CAC
TGF-*β*1 (rat) -F	GCT​GAA​CCA​AGG​AGA​CGG​AAT​A
TGF-*β*1 (rat) -R	GCA​GGT​GTT​GAG​CCC​TTT​CC
PI3K (rat) -F	AGC​TGG​TCT​TCG​TTT​CCT​GA
PI3K (rat) -R	GAA​ACT​TTT​TCC​CAC​CAC​GA
AKT (rat) -F	TCA​TTG​AGC​GCA​CCT​TCC​AT
AKT (rat) -R	CTC​CTG​CCG​TTT​GAG​TCC​AT
FN (rat) -F	AGG​CAC​AAG​GTC​CGA​GAA​GAG​G
FN (rat) -R	CAT​GAG​TCA​TCC​GTA​GGC​TGG​TTC
COL-I (rat) -F	GAC​AGG​CGA​ACA​AGG​TGA​CAG​AG
COL-I (rat) -R	TGC​AGG​ACC​AGA​AGC​TCC​AGA​G
α-SMA (rat) -F	GAA​CAC​GGC​ATC​ATC​ACC​AA
α-SMA (rat)-R	ATC​TCC​AGA​GTC​CAG​CAC​AAT​A

### 2.8 Western blot detection

For each experimental group, three biological replicates (animals) were selected for Western blot analysis. These animals were chosen based on their target protein levels measured by BUN, SCr and UTP to ensure representation of the group mean and to minimize outlier influence. The observed protein expression changes were highly reproducible across the three independent samples.

The expression levels of p-PI3K, PI3K, AKT, p-AKT, Collagen I, Collagen III, Fibronectin (Fn), MMP-9, TIMP-1, TGF-β1, SMAD3 and p-SMAD3 were detected by Western blot. The renal tissue was ground evenly and placed in a sterile centrifuge tube. RIPA lysis buffer was added. The renal tissue protein was extracted according to the kit instructions, and the protein concentration of each group was determined. Total protein 50 μg was mixed with Loading buffer and denatured at 100 °C for 5 min. After 2 h of 10% SDS-PAGE gel electrophoresis, PVDF membrane was transferred for 1 h. After incubation with skimmed milk powder for 1.5 h, the primary antibody (1:1,000) was added and incubated at 4 °C overnight. After incubation with secondary antibody (1:10,000) for 1 h, the gray value of each band was calculated by ImageJ software.

### 2.9 Molecular docking

Molecular docking was performed between the core targets related to chronic kidney injury and the main components in the AO. The compound small molecule ligand files in the AO were downloaded from the PubChem database (https://pubchem.ncbi.nlm.nih.gov) and output in MOL2 format. The corresponding PDB format files were downloaded from the RCSB PDB database (https://www.rcsb.org), including ITGAV (PDB ID: 4UM9), FAK (PDB ID: 2JKK), PI3K (PDB ID: 1E8Y) and AKT (PDB ID: 2UZT). The target proteins were pretreated by removing water molecules and ligands using Discovery Studio 4.5 software, and the compounds were docked with the targets in the software.

### 2.10 Statistical analysis

Statistical analysis was performed using GraphPad Prism 7.00 software. All data were expressed as mean ± standard deviation (x̄ ± s). Normality was assessed by Shapiro-Wilk test, and homogeneity of variance was verified by Levene’s test. For comparisons involving more than two groups, one-way ANOVA was used, followed by Tukey’s HSD *post hoc* test for multiple pairwise comparisons. *P* value <0.05 was considered statistically significant.

## 3 Results

### 3.1 The effect of *Amomum tsao-ko* on renal function

Compared with the sham group, the 24 h urine UTP content of rats in the 5/6 Nx group increased, and the serum BUN and SCr contents increased significantly (*P* < 0.01). After treatment with AO, it was found that the urine UTP content decreased significantly (*P* < 0.01), and the serum BUN and SCr contents decreased gradually with the increase of AO. There was no statistical significance in losartan group. The results are shown in Figure 1.

**FIGURE 1:**
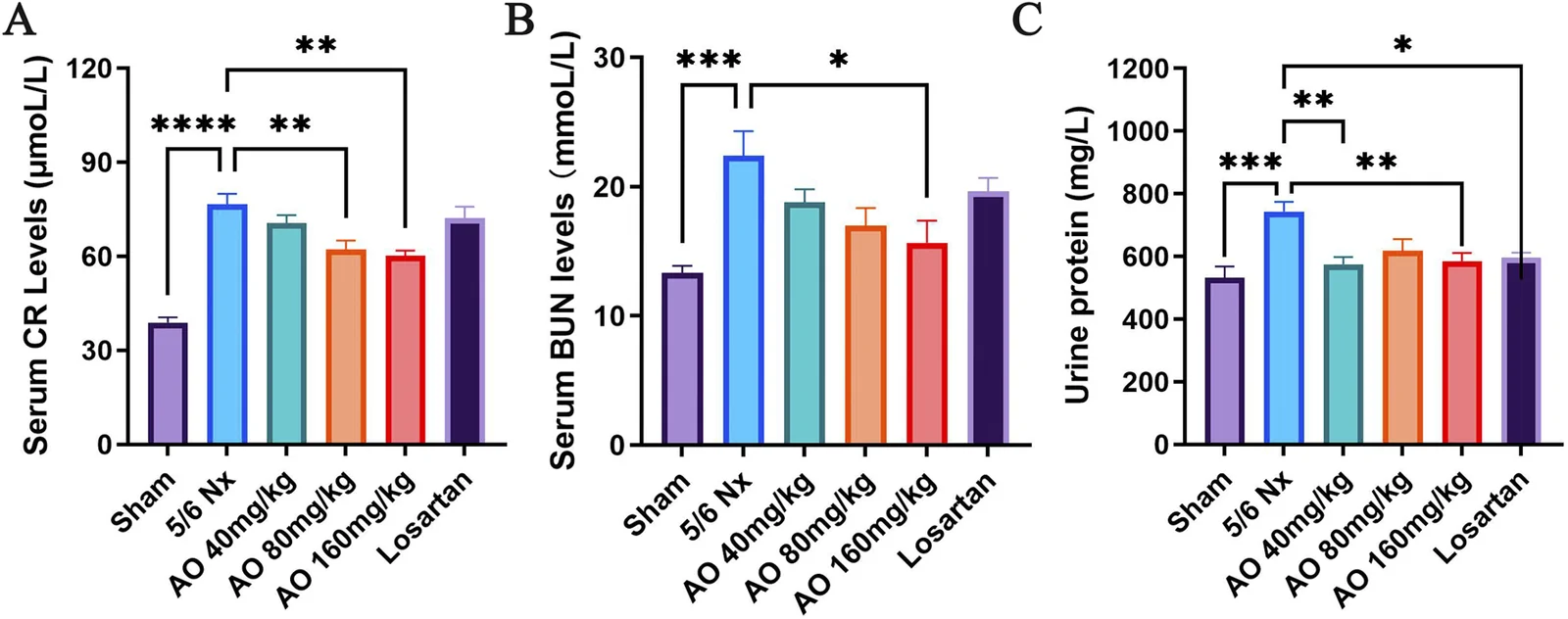
Detection results of renal injury indexes in rats of each group. **(A)** SCr test results; **(B)** BUN test results **(C)** UP test results. (mean ± SD, n = 8, ^*^ *P* < 0.05, ^**^ *P* < 0.01, ^***^ *P* < 0.001, ^****^ *P* < 0.0001).

### 3.2 Renal histopathological staining results

HE staining showed that the kidney morphology of the rats in the sham operation group was good, the glomerular structure was normal, the basement membrane was arranged well, and the capillaries were not narrowed or occluded. However, compared with the sham group, 5/6 Nx rats showed significant glomerulosclerosis, tubular atrophy, expansion and epithelial hyperplasia. With the increase of the dose of AO, the pathological damage of 5/6 Nx rats was significantly reduced.

PAS staining results showed that compared with the sham operation group, the gap between renal tubular epithelial cells in the 5/6 nephrectomy rat model group became larger, and a large number of inflammatory cells infiltrated in the interstitium. Renal tubular epithelial cells showed atrophy, deformation, shedding, and even exposed basement membrane. The treatment of AO can significantly reduce the infiltration of inflammatory cells.

Masson staining results showed that compared with the sham operation group, 5/6 Nx could develop extensive interstitial fibrosis, and the fibrosis phenomenon gradually decreased with the increase of the dose of AO. The results are shown in Figure 2.

**FIGURE 2:**
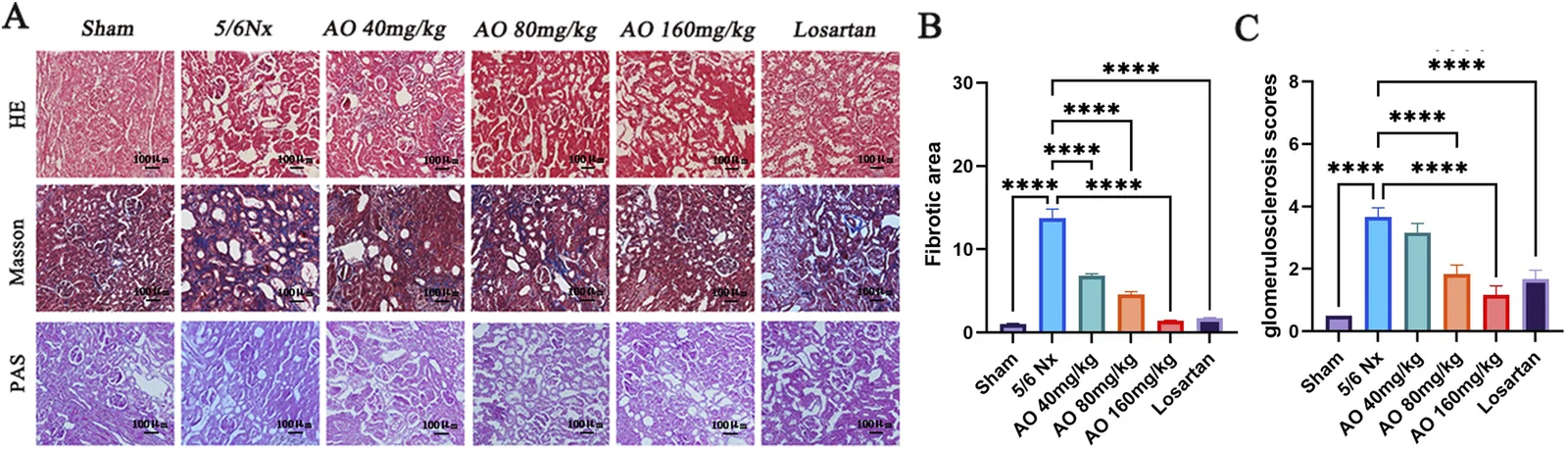
Histopathological assessment of renal injury. **(A)** Representative HE, Masson and PAS staining images (×200). **(B)** Renal fibrotic area. **(C)** Glomerulosclerosis scores. (mean ± SD, n = 3, ^*^ *P* < 0.05, ^**^ *P* < 0.01, ^***^ *P* < 0.001, ^****^ *P* < 0.0001).

### 3.3 RNA-seq

To further elucidate the impact of HB on the chronic renal fibrosis, RNA sequencing was performed on renal tissue samples. RNA sequencing analysis revealed that there were 823 genes with significant differences in expression between the 5/6 Nx group and the Sham group (|log FC|>2, adjusted *P <* 0.05), of which 396 genes were downregulated and 427 genes were upregulated, as shown in Figure 3A. Compared with the 5/6 Nx group, there were significant differences in the expression of 1,009 genes in the AO group, of which 503 genes were downregulated and 506 genes were upregulated, as shown in Figure 3B. The heat map showed the first 30 differential genes (Figures 3C,D). In order to further study the mechanism of CKD, these differentially expressed genes were enriched and analyzed by gene ontology (GO). GO was used to annotate 20 differentially expressed genes. GO annotation analysis showed that these genes were closely related to drug response, response to external biological stimulation, inflammatory response, cell migration, *etc.* (Figures 3E,F). KEGG pathway enrichment analysis revealed that the top four enriched pathways were ECM‑receptor interaction, Focal adhesion, PI3K‑Akt signaling pathway, and Pathways in cancer, suggesting that these pathways may be involved in the initiation and progression of renal fibrosis (Figure 4).

**FIGURE 3:**
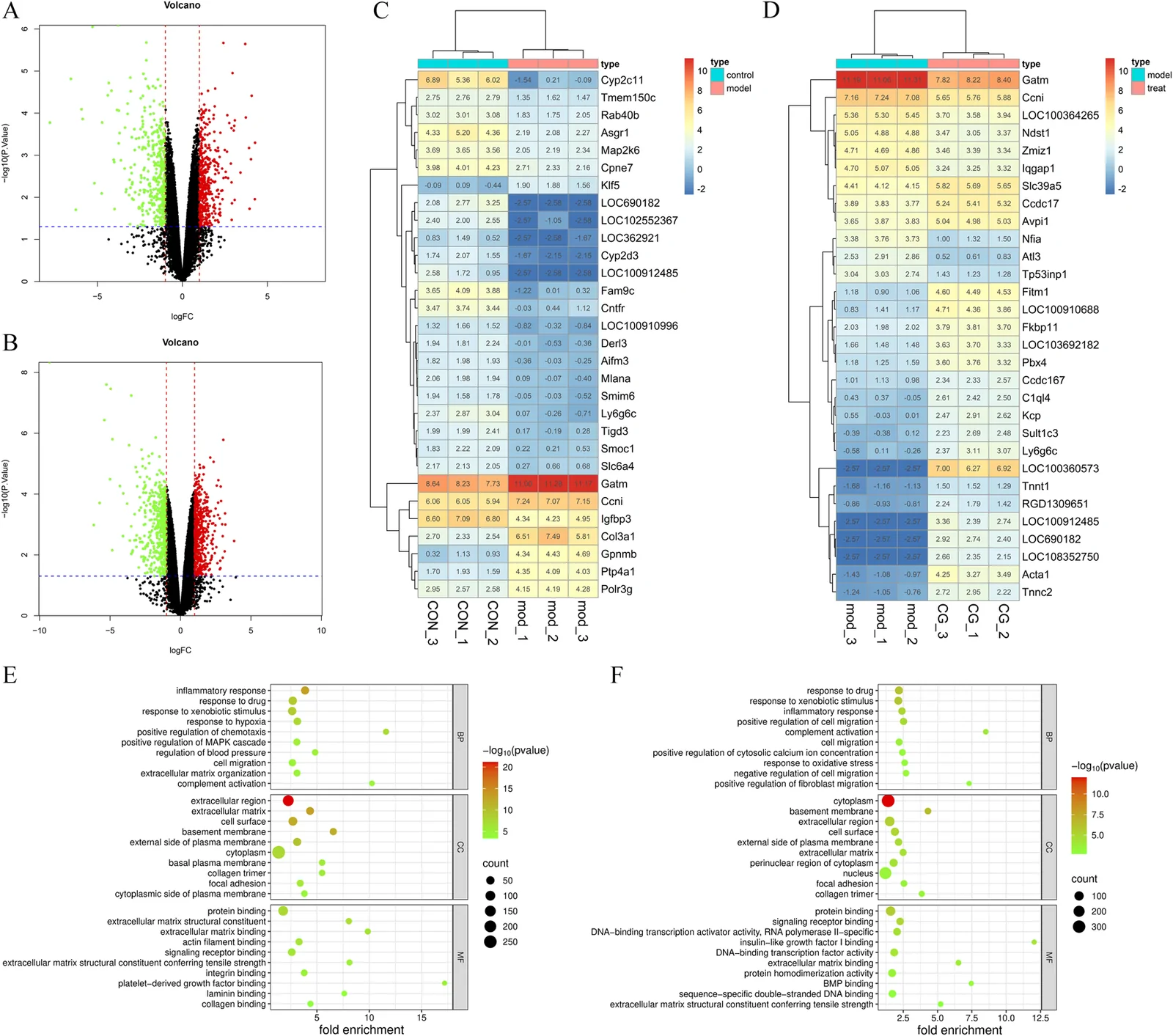
RNA seq analysis revealed differential transcriptome profiles in rat kidney tissue. **(A)** Volcano map shows the expression profile changes of the sham group and the 5/6 Nx group; **(B)** Volcano diagram showed the expression profile changes of 5/6 Nx group and AO treatment group. **(C)** Heat map showed the expression of upregulated or downregulated genes (Sham group and 5/6 Nx group). **(D)** Heat map showed upregulated or downregulated gene expression (5/6 Nx group and AO group). **(E)** GO enrichment analysis of differential genes in Sham group and 5/6 Nx group; **(F)** GO enrichment analysis of differential genes between 5/6 Nx group and AO group.

**FIGURE 4:**
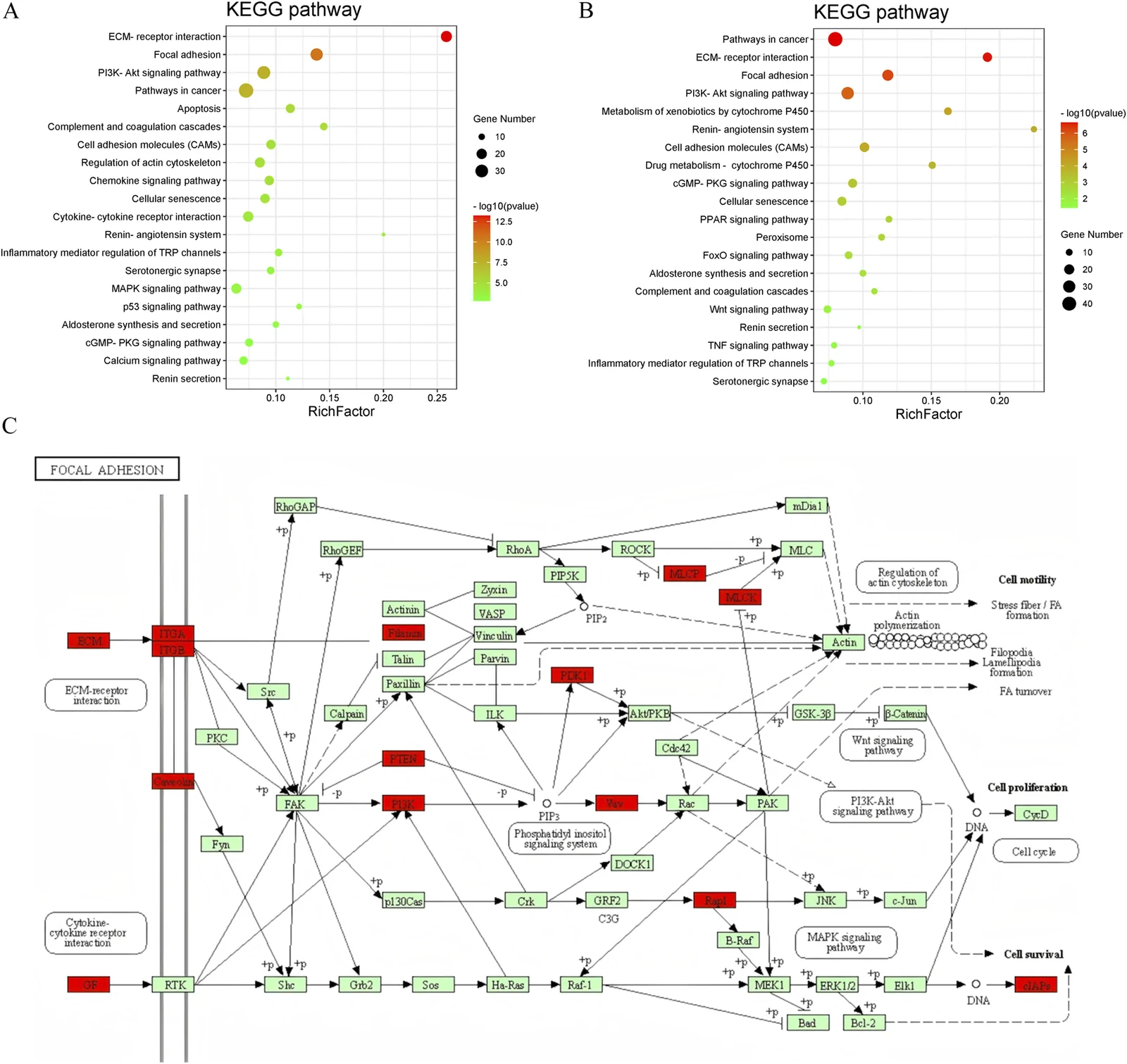
Pathway analysis of transcriptome in renal injury 5/6 Nx group and AO group. **(A)** KEGG pathway enrichment bubble plot of differential genes in Sham group and 5/6 Nx group; **(B)** KEGG pathway enrichment bubble plot of differential genes in 5/6 Nx group and AO group; **(C)** KEGG analysis of the effect of AO on signal pathway.

### 3.4 Molecular docking

Molecular docking was performed using Discover Studio 4.5 to further investigate the binding sites and binding modes between compounds in the AO and key target proteins. The PDB file was obtained from the RCSB protein database (http://www.rcsb.org/pdb/home/home.do). The results showed that the main components of eucalyptus oil, citral, and geraniol in the AO could stably bind to key target proteins such as ITGAV/B6, FAK, PI3K, and AKT. Therefore, AO may exert renal protective effects through ECM receptor interactions, adhesion kinase signaling pathways, and PI3K-AKT signaling pathways. The results are shown in Figure 5.

**FIGURE 5:**
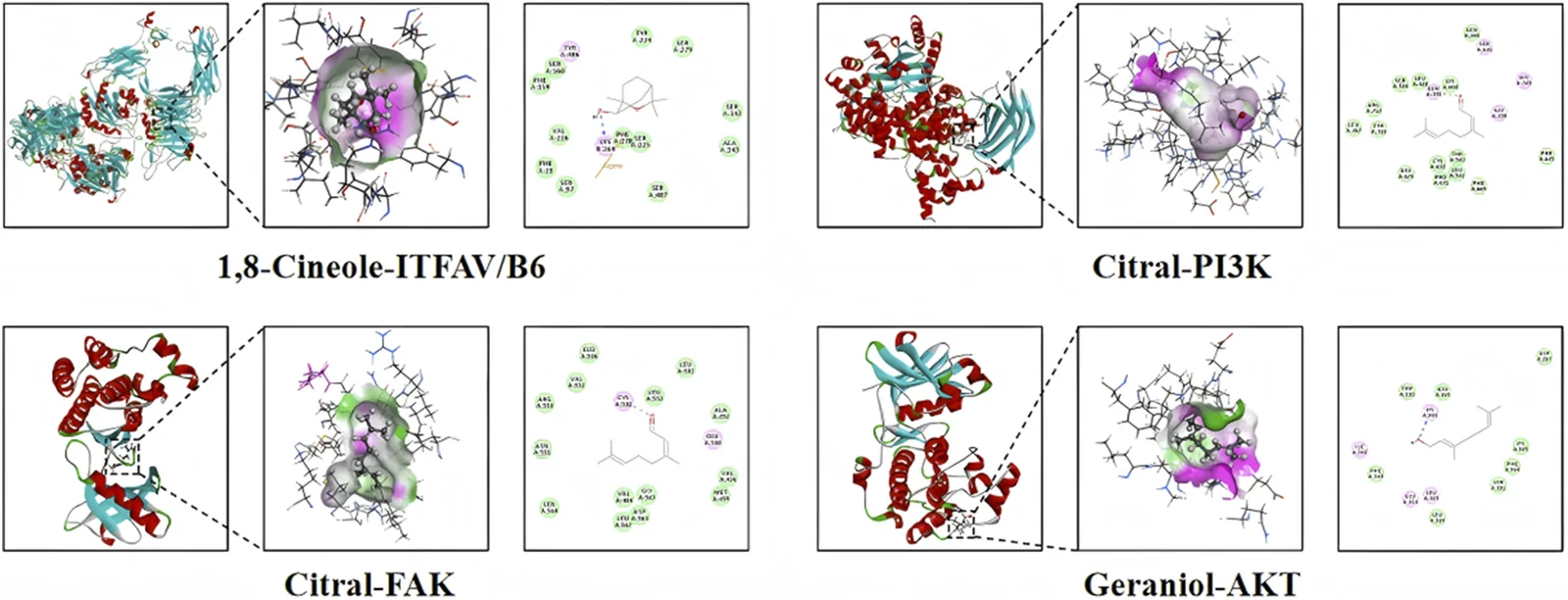
The binding of the main chemical components in the AO (1,8-Cineole, Citral, Geraniol) to key target proteins (ITFAV/B6, PI3K, FAK, AKT).

### 3.5 Effect of AO on extracellular matrix-related proteins in 5/6 nephrectomy rats

The results are shown in Figure 6. Compared with the Sham group, the expression of Collagen I, Collagen III, Fibronectin (Fn), α-SMA and TIMP-1 in the 5/6 Nx group was significantly increased, while the expression of MMP-9 and E-cadherin (E-CAD) was significantly decreased. With the increase of the AO dose, the expression of Collagen I, Collagen III, Fn, α-SMA and TIMP-1 gradually decreased, and the expression of MMP-9 and E-CAD gradually increased. In the high-dose AO group, these changes were statistically significant (*P* < 0.01). These results suggest that AO can inhibit myofibroblast activation and epithelial-mesenchymal transition (EMT), reduce ECM deposition, and thereby alleviate renal fibrosis. Next, we validated the identified differentially expressed genes using RT-qPCR, as shown in Figure 7.

**FIGURE 6:**
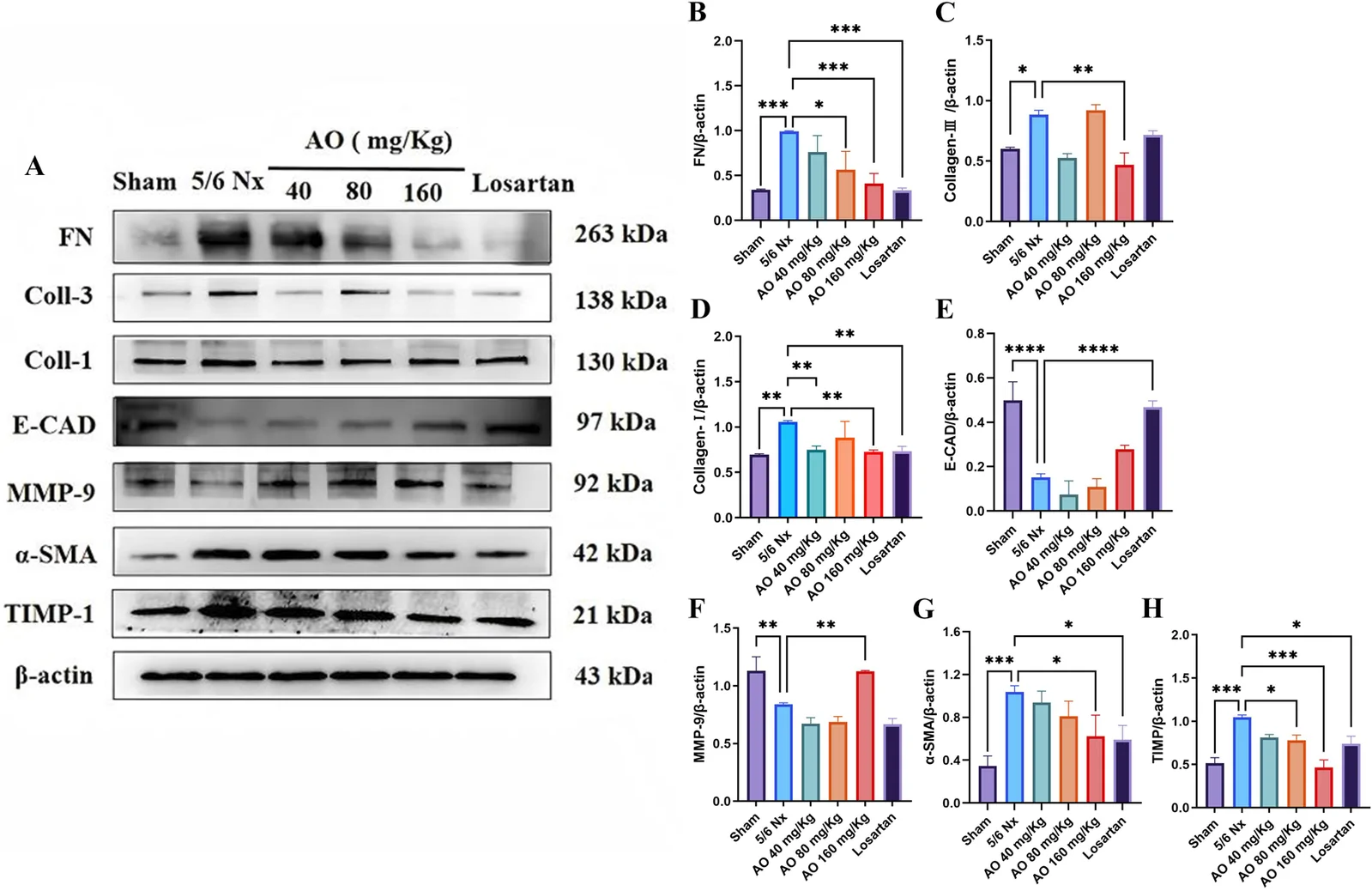
AO ameliorated renal fibrosis in 5/6 nephrectomized rats. **(A)** Representative Western blot bands of COL-1, FN, and α-SMA etc., in each group; **(B)** Relative expression of FN/β-actin. **(C)** Relative expression of Collagen-III/β-actin; **(D)** Relative expression of Collagen-I/β-actin. **(E)** Relative expression of E-CAD/β-actin; **(F)** Relative expression of MMP-9/β-actin. **(G)** Relative expression of α-SMA/β-actin; **(H)** Relative expression of TIMP/β-actin. (mean ± SD, n = 8, ^*^ *P* < 0.05, ^**^ *P* < 0.01, ^***^ *P* < 0.001, ^****^ *P* < 0.0001).

**FIGURE 7:**
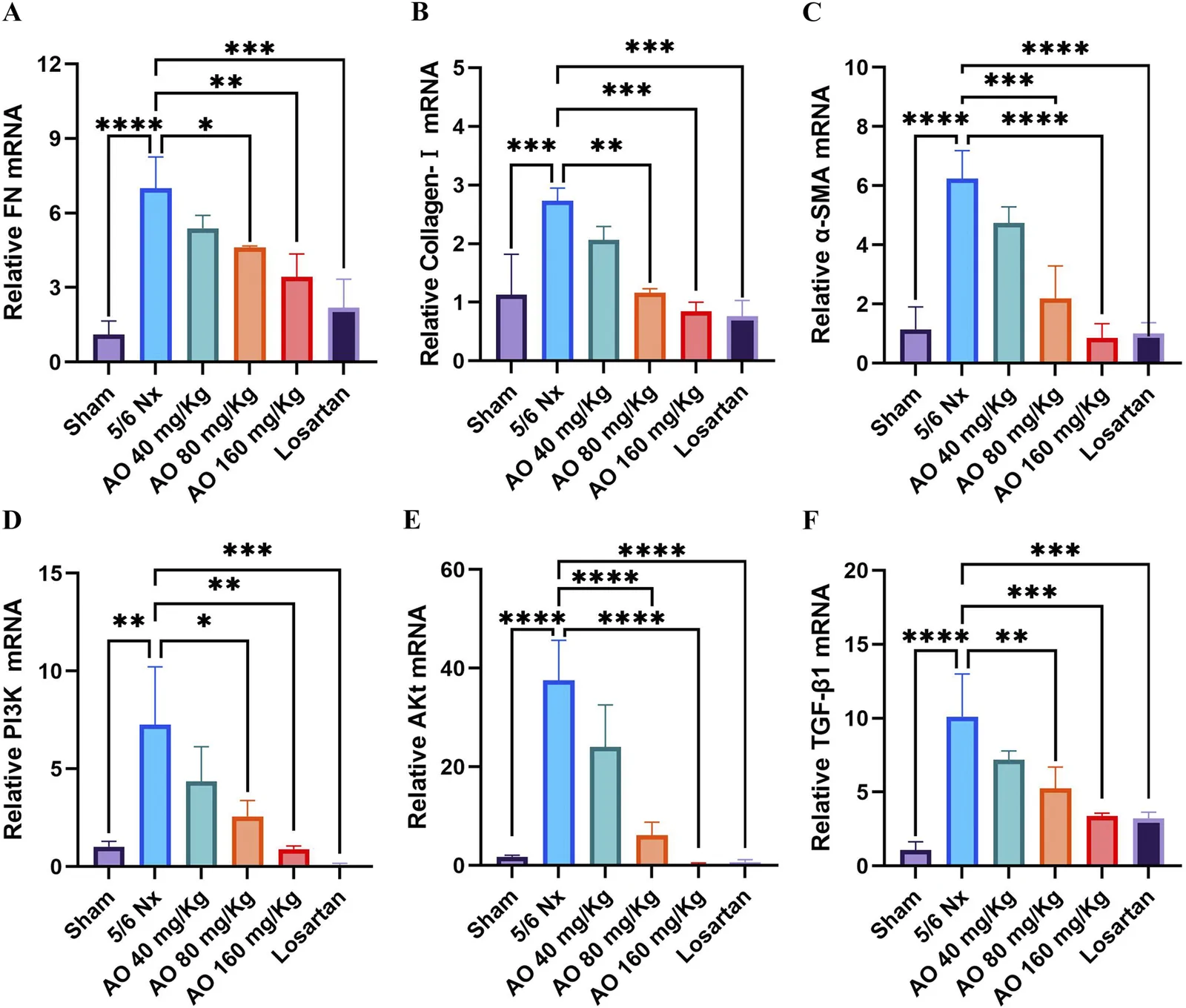
Verification of the differential genes by RT-qPCR. **(A)** Relative FN mRNA; **(B)** Relative Collagen-I mRNA. **(C)** Relative α-SMA mRNA; **(D)** Relative PI3K mRNA. **(E)** Relative AKT mRNA; **(F)** Relative TGF-*β*1 mRNA.

### 3.6 Effect of AO on PI3K/AKT and TGF-*β*1/SMAD3 signaling pathway in 5/6 nephrectomy rats

The results are shown in Figure 8 Compared with the Sham group, the phosphorylation level of PI3K in the renal tissue of the 5/6 Nx group was significantly increased (*P* < 0.01). With the increase of the dosage of AO, the expression of PI3K decreased gradually. The expression of PI3K was significantly decreased under the treatment of high dose of AO (*P* < 0.01). AO could significantly reduce the phosphorylation level of PI3K in renal tissue of 5/6 nephrectomy rats. Compared with the Sham group, the expression of TGF-β1 and p-SMAD3 protein in the 5/6 Nx group was significantly increased (*P* < 0.01). With the increase of the dose of AO, the expression of TGF-β1 and p-SMAD3 decreased gradually. The expression of TGF-β1 and p-SMAD3 decreased significantly under the treatment of high-dose AO. (*P* < 0.01). Next, we validated the identified differentially expressed genes using RT-qPCR, as shown in Figure 7.

**FIGURE 8:**
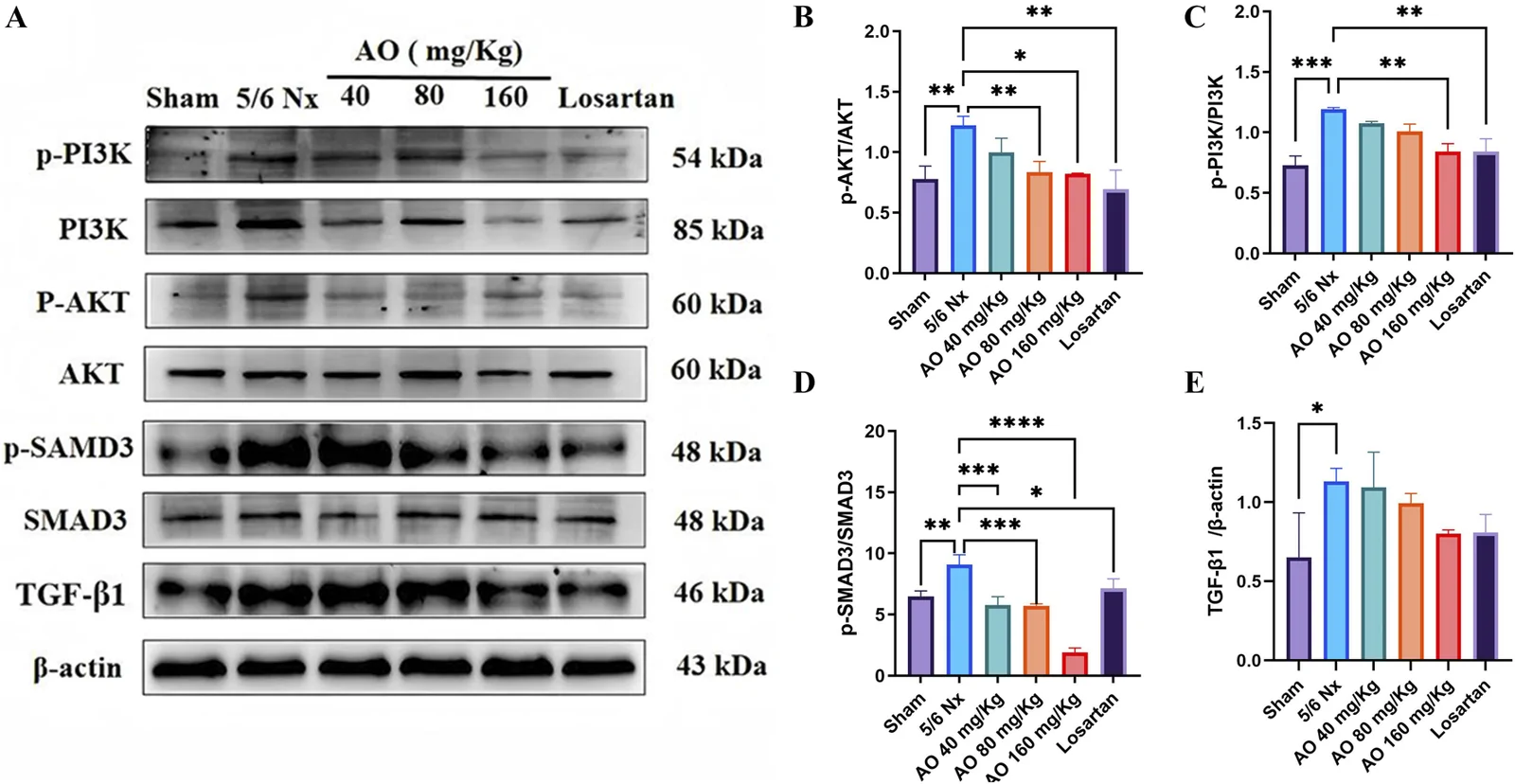
AO regulated PI3K/AKT and TGF-*β*1/SMAD3 signaling pathways in 5/6 nephrectomized rats. **(A)** Representative Western blot bands of PI3K, AKT, and SMAD3, etc., in each group; **(B)** Relative expression of p-AKT/AKT. **(C)** Relative expression of p-PI3K/PI3K; **(D)** Relative expression of p-SMAD3/SMAD3. **(E)** Relative expression of TGF-*β*1/β-actin. (mean ± SD, n = 8, ^*^ *P* < 0.05, ^**^ *P* < 0.01, ^***^ *P* < 0.001, ^****^ *P* < 0.0001).

## 4 Discussion

Renal fibrosis is an irreversible common pathological endpoint of CKD progression to end-stage renal disease (ESRD), which is characterized by abnormal deposition of ECM in renal tissue and progressive destruction and loss of function of normal renal structure (Niculae et al., 2023; Beran et al., 2025). ACEI and other drugs are used clinically to control renal fibrosis, but the effect is not ideal (Huang et al., 2025). In this study, we investigated the therapeutic potential of *Amomum tsao-ko* on chronic kidney injury. In a 5/6 nephrectomy rat model, AO could significantly inhibit the activation of TGF-*β*1/SMAD3 and PI3K/AKT signaling pathways, reduce extracellular matrix deposition, alleviate renal interstitial fibrosis, improve renal function, and thus treat chronic kidney injury.

The interaction of ECM receptors is closely related to the occurrence and development of chronic kidney injury, and its main components are type I and type III collagen fiber proteins (Balaji Ganesh et al., 2025). MMPs are a family of endopeptidases with strict regulatory mechanisms. They play a major role in CKD by remodeling and degrading ECM components (Sun et al., 2025). MMP-9 mainly degrades gelatin, type I and type III collagen, and laminin. It is a key degrading enzyme for extracellular matrix proteins (Song et al., 2024). The TIMP family is the core molecule that regulates ECM metabolism (Coates-Park et al., 2024). It maintains the dynamic balance of ECM synthesis and degradation by specifically inhibiting the activity of MMPs (Cabral-Pacheco et al., 2020). TIMP-1 can significantly inhibit the activity of MMP-9, and its overexpression will cause excessive accumulation of extracellular matrix. TGF-*β*1 stimulates the activation and proliferation of fibroblasts and increases ECM synthesis. It is currently the most studied cytokine in the field of renal fibrosis and is also recognized as a pro-fibrotic factor (Oftring et al., 2025). Our research indicate that the treatment with AO alleviates the inhibitory effect of TIMP-1 on MMP-9 and reduces the expression of ECM receptor related proteins, thereby improving the treatment of chronic kidney injury in renal interstitial fibrosis. Beyond ECM regulation, renal fibrosis is also critically dependent on pro-fibrotic M2 macrophage activation. In this context, the histone demethylase JMJD3 (An et al., 2023) and the transcription factor STAT6 (Jiao et al., 2021) have been identified as indispensable drivers of M2 polarization, and both operate downstream of, or in parallel with, PI3K/AKT signaling, suggesting that the pathway alterations observed here may contribute to ECM deposition partly through macrophage-mediated mechanisms.

TGF-*β*1 induced phosphorylation of SMAD3 directly regulates the expression of α-SMA and ECM proteins such as collagen and fibronectin, which are classic markers of myofibroblasts. The expression of TGF-*β*1 signaling increases the degree of renal fibrosis in CKD patients and animals (Goncalves et al., 2021). The PI3K-AKT pathway has received extensive attention in chronic kidney disease in recent years because of its ability to regulate collagen metabolism (Yang et al., 2025). Studies have shown that PI3K can form a positive feedback loop with the TGF-*β*1 pathway to jointly regulate ECM deposition. TGF-*β*1 is considered to be a key driver of fibrosis, and its type I receptor (T*β*RI) can directly phosphorylate PI3K, regulate subunit p85, and block its nuclear translocation to relieve the inhibition of anti-fibrosis genes (Wang et al., 2026). PI3K activates the downstream Akt/mTOR axis by catalyzing phosphatidylinositol-4,5-diphosphate, regulates cell proliferation, survival and anabolism, and maintains the dynamic balance of ECM synthesis and degradation under normal conditions (Fang et al., 2020). In chronic renal fibrosis, overexpression of TGF-*β*1 stimulates PI3K p110α subunit, leading to continuous phosphorylation of Akt, further inhibiting collagen degradation and promoting transcription of Collagen I and Collagen III. The significant expression of P-PI3K and P-AKT in the experimental results not only confirmed the activation of PI3K-AKT pathway in the pathological process of CKD, but also suggested that the regulation of upstream and downstream proteins of PI3K-AKT pathway by intervention may be an effective way to treat CKD and reduce renal injury (Shao et al., 2025; Yuan et al., 2026; Li et al., 2023). Additionally, the PI3K/AKT cascade is subject to negative regulation by PTEN, and PTEN suppression has been linked to aggravated fibrotic remodeling (An et al., 2022). Moreover, downstream ECM transcription is governed by mechanotransducers such as TEAD1 (Tran et al., 2025) and pro-fibrotic factors like SOX4 (Du et al., 2026), both of which engage in extensive crosstalk with the TGF-*β*1/SMAD3 axis. The TGF-*β*1/SMAD3 pathway and the PI3K-AKT pathway jointly regulate the differentiation of integrated fibroblasts and ECM protein expression, playing an important role in the progression of renal fibrosis.

Metabolic stress, a hallmark of the uremic milieu, has been recognized as a potent trigger of the cGAS-STING pathway (Jiao et al., 2025), which in turn synergizes with NLRP3 inflammasome activation to sustain chronic renal inflammation. Given that PI3K/AKT is intimately linked to cellular metabolic sensing, the pathway alterations observed in our 5/6 Nx model may represent an upstream metabolic checkpoint that primes this cascade. A series of recent studies have confirmed that TGF-*β*1/SMAD3 and PI3K/AKT pathways function as critical upstream regulators of NLRP3 inflammasome-driven renal inflammation during glomerulosclerosis and CKD development. TGF-*β*1/SMAD3 priming and PI3K/AKT hyperactivation synergistically promote NLRP3 inflammasome activation and pro-inflammatory cytokine secretion, jointly aggravating renal fibrotic lesions. While this work mainly dissects the fibrotic functions of TGF-*β*1/SMAD3 and PI3K/AKT, we acknowledge the central role of the NLRP3 inflammasome in linking inflammation and fibrosis. Further investigations will focus on exploring NLRP3 inflammasome activity in our experimental system, so as to complete the full molecular cascade governing CKD and glomerular sclerosis (Pang et al., 2022; Datta et al., 2025; Ji et al., 2024).

In summary, this study found that in the 5/6 Nx model, there is activation of the TGF-*β*1/SMAD3 and PI3K/AKT signaling pathways, accompanied by ECM deposition to aggravate the process of renal fibrosis. As a treatment group drug, AO improved the above fibrosis indicators, suggesting that AO may alleviate ECM deposition by regulating TGF-*β*1/SMAD3 and PI3K/AKT signaling pathways to alleviate CKD renal fibrosis injury, thereby delaying CKD progression (Figure 9).

**FIGURE 9:**
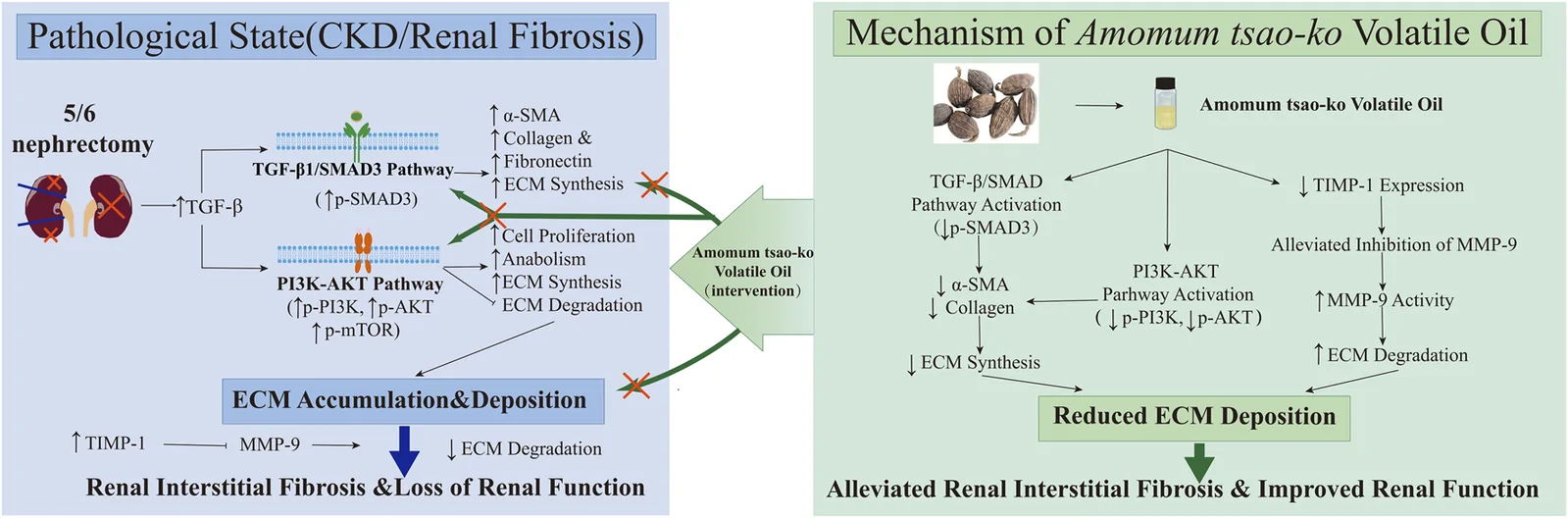
Prediction of mechanism of CKD induced by 5/6 nephrectomy in rat.

## 5 Conclusion

Based on the long-standing application of *Amomum tsao-ko* in the treatment of edema and renal dysfunction in traditional medicine, in this study, the therapeutic effect of AO on renal fibrosis may be related to regulating the levels of related proteins in the TGF-*β*1/SMAD3 and PI3K/AKT signaling pathways, and reducing extracellular matrix deposition. These findings provide a mechanism verification for the ethnic medicinal practice of *Amomum tsao-ko* in kidney diseases, and reveal how its bioactive components transform traditional efficacy into modern pharmacological effects, providing a new clinical example.

## Data Availability

The original contributions presented in the study are publicly available. This data can be found here: https://www.ncbi.nlm.nih.gov/bioproject/PRJNA1527663.
